# Influence of enrollment sequence effect on observed outcomes in the ADDRESS and PROWESS studies of drotrecogin alfa (activated) in patients with severe sepsis

**DOI:** 10.1186/cc7011

**Published:** 2008-09-11

**Authors:** Pierre-François Laterre, William L Macias, Jonathan Janes, Mark D Williams, David R Nelson, Amand RJ Girbes, Jean-François Dhainaut, Edward Abraham

**Affiliations:** 1 St Luc University Hospital, Avenue Hippocrate 10, 1200 Brussels, Belgium; 2 Lilly Research Laboratories, Eli Lilly and Company, Indianapolis, IN 46285, USA; 3 Eli Lilly, Erl Wood Manor, Windlesham, Surrey GU20 6PH, UK; 4 Department of Intensive Care, VU University Medical Center, De Boelelaan 1105, 1081 HVAmsterdam, The Netherlands; 5 Paris Descartes University, rue de l'Ecole de Médecine, 75270 Paris Cedex 06, Paris, France; 6 University of Alabama at Birmingham School of Medicine, 1530-3^rd ^Avenue South, FOT 1203, Birmingham, AL 35294, USA

## Abstract

**Introduction:**

We performed a study to determine whether an enrollment sequence effect noted in the PROWESS (recombinant human activated Protein C Worldwide Evaluation in Severe Sepsis) trial exists in the ADDRESS (Administration of Drotrecogin Alfa [Activated] [DrotAA] in Early Stage Severe Sepsis) trial.

**Methods:**

We evaluated prospectively defined subgroups from two large phase 3 clinical trials: ADDRESS, which included 516 sites in 34 countries, and PROWESS, which included 164 sites in 11 countries. ADDRESS consisted of patients with severe sepsis at low risk of death not indicated for treatment with DrotAA. PROWESS consisted of patients with severe sepsis with one or more organ dysfunctions. DrotAA (24 μg/kg per hour) or placebo was infused for 96 hours.

**Results:**

In ADDRESS and PROWESS, there was a statistically significant interaction between the DrotAA treatment effect and the sequence in which patients were enrolled. In both trials, higher mortality was associated with DrotAA use in the subgroup of patients enrolled first at study sites. Compared with placebo, PROWESS mortality was lower with DrotAA treatment for the second and subsequent patients enrolled, whereas in ADDRESS, mortality remained higher for the second patient enrolled but thereafter was lower for DrotAA-treated patients. Comparison of patients enrolled first with subsequent patients enrolled indicated that the characteristics of patients changed. Subsequently enrolled patients were treated earlier, were less likely to suffer nonserious bleeds (ADDRESS), and experienced fewer protocol violations (PROWESS).

**Conclusions:**

Analyses suggest that an enrollment sequence effect was present in the ADDRESS and PROWESS trials. Analysis of this effect on outcomes suggests that it is most apparent in patients at lower risk of death. In PROWESS, this effect appeared to be associated with a reduction of the DrotAA treatment effect for the first patients enrolled at each site. In ADDRESS, this effect may have contributed to early termination of the study. The finding of an enrollment sequence effect in two separate trials suggests that trial designs, site selection and training, data collection and monitoring, and statistical analysis plans may need to be adjusted for these potentially confounding events.

**Trial Registration:**

ADDRESS trial registration number: NCT00568737. PROWESS was completed before trial registration was required.

## Introduction

The Protein C Worldwide Evaluation in Severe Sepsis (PROWESS) study demonstrated that drotrecogin alfa (activated) (DrotAA) reduced mortality in patients with severe sepsis [1]. Subgroup analyses suggested heterogeneity in the observed treatment effect for some subgroups, including those defined by baseline Acute Physiology and Chronic Health Evaluation (APACHE) II score, by protocol violation status, and by the sequence of enrollment at a study site [2,3]. Within these subgroups, the observed reduction in mortality associated with DrotAA was larger for patients with higher APACHE II scores, with no violation of the protocol, and who comprised the second and subsequent patients enrolled at a study site [3]. The latter two observations suggested that a learning curve appeared to be present within PROWESS such that the ability to demonstrate efficacy improved with increasing site experience with the study protocol [3].

Based on subgroup analyses of PROWESS, regulatory agencies approved the use of DrotAA in patients at higher risk of death as defined, for example, by an APACHE II score of greater than or equal to 25 or multiple-organ dysfunction (MOD) [4,5]. As a condition for approval, the US Food and Drug Administration required the sponsor to conduct a randomized placebo-controlled trial of DrotAA in the nonindicated population of severe sepsis patients at lower risk of death (the Administration of Drotrecogin Alfa [Activated] in Early Stage Severe Sepsis [ADDRESS] study) [6,7]. Based on the estimated placebo mortality rate in this lower-severity population, the ADDRESS study planned to enroll approximately 11,400 severe sepsis patients at 1,000 investigative sites in 35 countries. The ADDRESS study was prematurely terminated at the recommendation of the safety monitoring board because of a low likelihood of meeting the prospectively defined objective of demonstrating a significant reduction in the risk of 28-day all-cause mortality with DrotAA [7].

As a potential learning curve was present in the PROWESS trial and because the ADDRESS trial would require approximately 1,000 investigative sites, many of which were without prior clinical trial experience, prospectively defined analyses were included in the ADDRESS statistical analysis plan to assess the influence of any learning curve on the observed outcomes. We report the results of these analyses and additional exploratory analyses of both the PROWESS and ADDRESS databases. We discuss the results of these analyses in the context of their implication on the design and conduct of future clinical trials in patients with severe sepsis.

## Materials and methods

Both PROWESS and ADDRESS were randomized double-blind placebo-controlled studies evaluating the efficacy (28-day mortality) of DrotAA (Xigris^®^; Eli Lilly and Company, Indianapolis, IN, USA) given as an intravenous infusion (24 μg/kg per hour) for 96 hours in patients with severe sepsis. Both studies were approved by the ethics committee of each individual participating center, and written informed consent was obtained from each patient or next of kin. In PROWESS, patients were at a greater risk of death [1] than in ADDRESS [7] (placebo 28-day mortality 30.8% versus 17.0%, respectively). For ADDRESS, the study enrolled patients with severe sepsis not indicated for treatment with DrotAA under the applicable label in the country in which the patient was enrolled. Severe sepsis was defined as the presence of a known or suspected infection and at least one sepsis-induced organ dysfunction. The population indicated for DrotAA varied from country to country but was generally defined as patients with severe sepsis with MOD and/or an APACHE II score of greater than or equal to 25. Randomization was stratified by site and within site by heparin use.

### Statistical analyses

In the PROWESS study, the prospectively defined analysis to assess the influence of site enrollment on the observed treatment effect was an analysis of treatment effects that potentially differed across subgroup strata (using Breslow-Day tests). Potential interactions were identified for subgroups defined by presence versus absence of a significant protocol violation (*P *= 0.07), original versus amended protocol (*P *= 0.08), and APACHE II quartile at baseline (*P *= 0.09). Further examination of these interactions led to *post hoc *analyses of within-site sequence effects, as previously described [3]. Based on the *post hoc *significance of an interaction related to sequence, ADDRESS included a prospectively defined analysis to assess the influence of site enrollment on the observed treatment effect which was an analysis of 28-day mortality in the subgroups of first patients enrolled at each investigative site compared with the second and subsequent patients enrolled at each site (using Breslow-Day tests). The treatment effect was further assessed by analysis of mortality by the number of patients (1 to 4, 5 to 8, 9 to 12, and more than 12 patients) enrolled per site. Chi-square tests were used to compare mortality rates between treated and placebo patients.

## Results

At the time of termination, 2,640 patients had been enrolled in the ADDRESS study at 516 centers in 34 countries. Mortality data at day 28 were available for 2,613 patients (placebo, n = 1,297; DrotAA, n = 1,316). There was no statistical difference between the placebo and DrotAA groups in 28-day all-cause mortality (placebo, 17.0%; DrotAA, 18.5%; *P *= 0.34) (Table 1). Based on a prospectively defined analysis, there was a significant treatment-by-sequence of enrollment interaction for the first patient enrolled at each site compared with all subsequently enrolled patients at that site (*P = *0.04). Mortality at 28 days was higher for DrotAA patients compared with placebo patients in the subgroup of patients who comprised the first patients enrolled at each study site (22.3% versus 14.5%). Mortality rates were similar between treatment groups for the second and subsequent patients enrolled at each study site. Similar to what had previously been reported for PROWESS [3], treatment effect assessed by enrollment sequence grouped by block size (four patients per block) is listed in Table 2. In ADDRESS, mortality was higher for DrotAA patients compared with placebo in the first block, similar to placebo in the second block, and lower than placebo in the third and subsequent blocks. The median number of patients enrolled per site was eight. A treatment-by-enrollment sequence was observed at both small (≤8 patients) and high (>8 patients) enrolling sites (data not shown).

**Table 1:** Twenty-eight-day mortality for all patients enrolled in ADDRESS and for sequence subgroups

	Drotrecogin alfa (activated)		Placebo		Relative risk	95% CI	Breslow-Day *P *value
	Number	Died (percentage)	Number	Died (percentage)			

All randomly assigned patients	1,316	243 (18.47)	1,297	221 (17.04)	1.08	0.92, 1.28	
Patient classification							0.04
First patient only	260	58 (22.31)	249	36 (14.46)	1.54	1.06, 2.25	
Excluding first patient	1,056	185 (17.52)	1,048	185 (17.65)	0.99	0.82, 1.19	

ADDRESS, ADministration of DRotrecogin alfa (activated) in Early Stage Severe Sepsis; CI, confidence interval.

In PROWESS, a statistically significant treatment-by-enrollment interaction was also observed (*P *= 0.007) (Table 2). However, in PROWESS, mortality was lower for DrotAA patients compared with placebo in all randomization blocks, although the difference was larger in the second and subsequent blocks of patients. The relative risk associated with DrotAA was similar between ADDRESS and PROWESS for patients enrolled in the third and subsequent blocks. Additionally, patients enrolled in the third and subsequent blocks represented 45.5% of all PROWESS patients (n = 769/1,690) and only 21.4% of ADDRESS patients (n = 558/2,613).

**Table 2:** Mortality rates and relative risks for drotrecogin alfa (activated) by enrollment sequence within a site: ADDRESS and PROWESS

	ADDRESS					PROWESS				
	
Enrollment sequence within a site	Placebo		DrotAA			Placebo		DrotAA		
	
	Number	Mortality percentage	Number	Mortality percentage	RR (95% CI)	Number	Mortality percentage	Number	Mortality percentage	RR (95% CI)
1st to 4th patients	727	15.5%	741	20.1%	1.29 (1.04, 1.62)	279	31.5%	280	28.6%	0.91 (0.71, 1.17)

5th to 8th patients	303	17.5%	284	17.3%	0.99 (0.69, 1.40)	179	29.6%	183	24.4%	0.81 (0.58, 1.14)

9th to 12th patients	132	19.7%	142	16.2%	0.82 (0.49, 1.37)	128	25.8%	121	20.7%	0.80 (0.51, 1.26)

>12th patients	135	21.5%	149	14.8%	0.69 (0.42, 1.14)	254	33.5%	266	22.9%	0.69 (0.52, 0.91)

ADDRESS, ADministration of DRotrecogin alfa (activated) in Early Stage Severe Sepsis; CI, confidence interval; DrotAA, drotrecogin alfa (activated); PROWESS, Protein C Worldwide Evaluation in Severe Sepsis; RR, relative risk.

**Figure 1:**
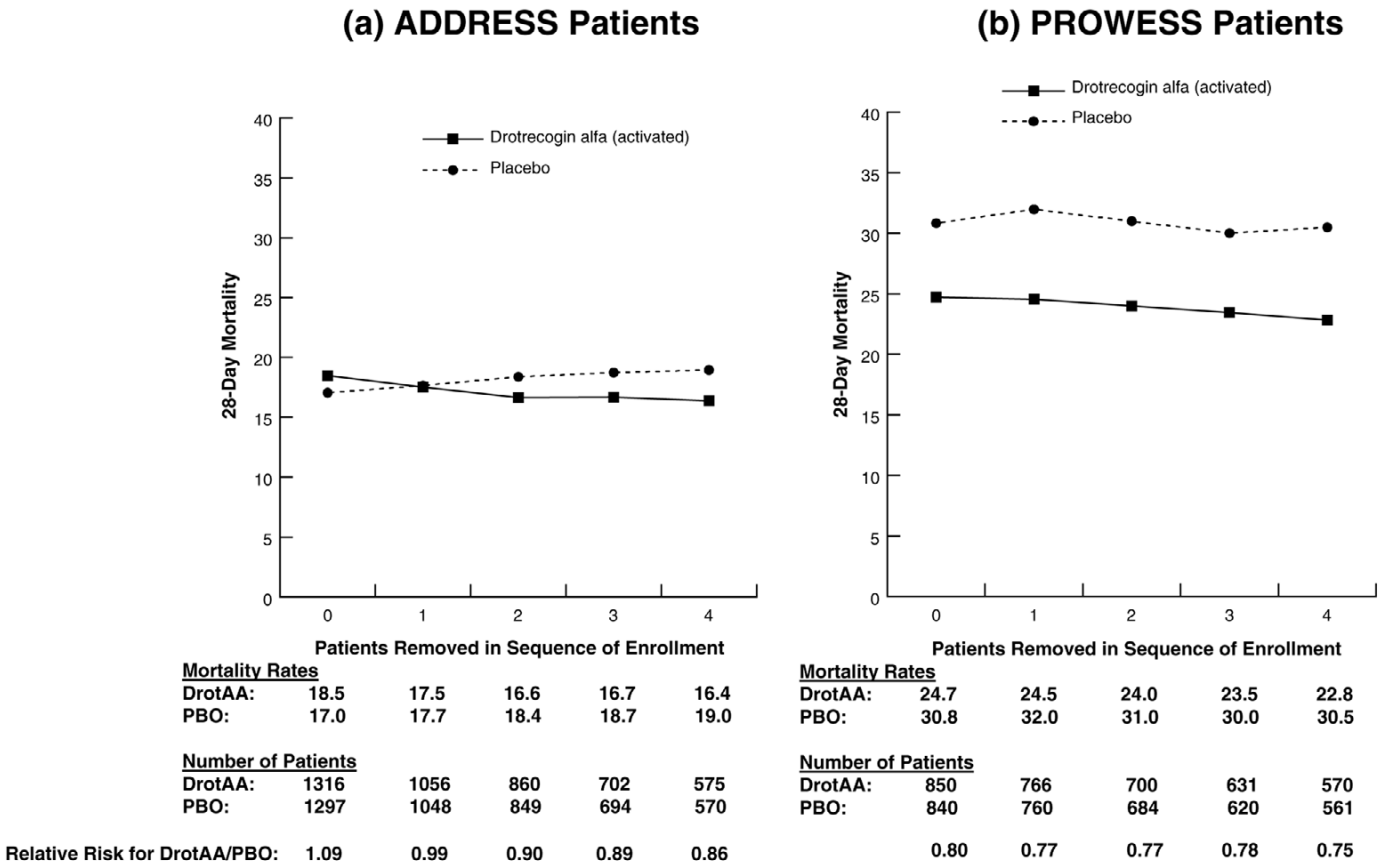
Twenty-eight-day mortality in all randomly assigned patients in ADDRESS **(a) **and PROWESS **(b) **with no patients removed from the analysis and also with the first through fourth patients enrolled at each site removed. Note: 0 represents the results for the entire population, and 1 through 4 correspond to the analysis with the first through fourth patients from each site removed. ADDRESS, ADministration of DRotrecogin alfa (activated) in Early Stage Severe Sepsis; DrotAA, drotrecogin alfa (activated); PBO, placebo; PROWESS, Protein C Worldwide Evaluation in Severe Sepsis.

In PROWESS, randomization was stratified only by site, resulting in a uniform block size of four at each site. However, in ADDRESS, randomization was also stratified by baseline heparin use, so there was no uniform block size for randomization, thus the first four patients (in theory) could have all received DrotAA or all placebo or some other combination. Thus, further exploratory analyses were performed by subgroups in which the first through fourth patients enrolled at each site were excluded from the analysis. In ADDRESS (Figure 1a), 28-day mortality was lower for DrotAA patients compared with placebo patients in the subgroup of patients excluding the first two patients enrolled at a site (16.6% versus 18.4%). These data are similar to those in PROWESS in which higher mortality was observed for DrotAA patients compared with placebo patients who comprised the first enrolled patients at each site (n = 164 patients, 26.2% versus 20.0%). However, this 'first patient' effect was relatively small compared with the remaining patients in the study (Figure 1b).

To explore interactions between mortality and the sequence of patient enrollment, ADDRESS patients were divided into subgroups comprising the first two enrolled patients at each site (≤2 subgroup, n = 904) and those comprising the third and subsequently enrolled patients (≥3 subgroup, n = 1,709). For patients in the ≤2 subgroup, 28-day mortality rates were 21.9% and 15.8% for DrotAA and placebo patients, respectively. In the ≥3 subgroup, 28-day mortality rates were 16.6% and 18.4% for DrotAA and placebo patients, respectively. Baseline characteristics for these subpopulations are shown in Table 3. Compared with patients in the ≤2 subgroup, patients in the ≥3 subgroup site tended to be enrolled in countries other than the US and Canada (indicating that 'patients per site' rates were generally lower in the US and Canada compared with the rest of the world), had lower acute physiology scores, and were more likely to have chronic health points, to have undergone recent surgery, and to have received prophylactic-dose heparin. These patients were also less likely to have community-acquired infections. Additionally, time from documented first organ dysfunction to start of study drug administration was shorter for third and subsequent patients enrolled at a site compared with the first two patients enrolled. In PROWESS, approximately 90% of patients started study drug within 24 hours [1], whereas in ADDRESS this was only 50%.

**Table 3:** Baseline characteristics for ADDRESS sequence subgroups

Variable	First 2 patients (n = 916)	3rd and subsequent patients (n = 1,724)	*P *value
Male, number (percentage)	517 (56.4%)	999 (57.9%)	0.46
Age in years, mean ± SD	58.3 ± 16.8	58.9 ± 16.6	0.36
Region, number (percentage)			<0.001
Europe	265 (28.9%)	581 (33.7%)	
US and Canada	480 (52.4%)	681 (39.5%)	
Other countries	171 (18.7%	462 (26.8%)	
Racial origin, number (percentage)			0.001
African descent	67 (7.3%)	98 (5.7%)	
Caucasian	694 (75.8%)	1,221 (70.8%)	
Hispanic	57 (6.2%)	144 (8.4%)	
Asian	62 (6.7%)	168 (9.7%)	
Other	36 (3.9%)	93 (5.4%)	
Number of organ failures, mean ± SD	1.5 ± 0.7	1.4 ± 0.7	0.19
APACHE II score, mean ± SD	18.3 ± 5.7	18.1 ± 5.9	0.22
Acute physiology score, mean ± SD	14.1 ± 5.4	13.7 ± 5.4	0.07
Number of patients with chronic health points (percentage)	221 (24.1%)	476 (27.6%)	0.08
Recent surgery, number (percentage)	321 (35.0%)	681 (39.5%)	0.06
Time from first organ failure to start of study drug in hours, mean ± SD	24.1 ± 13.6	21.8 ± 13.7	<0.001
Community-acquired infection, number (percentage)	688 (75.1%)	1,235 (71.6%)	0.06
Heparin use at baseline, number (percentage)	504 (55.0%)	1,047 (60.7%)	0.005

Frequencies were analyzed using a chi-square test. Means were analyzed using a type III sum squares analysis of variance on the ranks. ADDRESS, ADministration of DRotrecogin alfa (activated) in Early Stage Severe Sepsis; APACHE, Acute Physiology and Chronic Health Evaluation; SD, standard deviation.

The frequencies of serious bleeding events and any bleeding events in ADDRESS were also compared between the ≤2 and ≥3 subgroups (Table 4). The frequencies of serious bleeding events were similar between the ≤2 and ≥3 subgroups for both DrotAA and placebo patients. For DrotAA patients, a statistically significantly higher percentage of patients in the ≤2 subgroup experienced 'any bleeding' and 'any bleeding during the infusion' compared with DrotAA patients in the ≥3 subgroup. A lower percentage of DrotAA patients in the ≥3 subgroup experienced a transfusion compared with patients in the ≤2 subgroup (*P *= 0.13). A similar pattern for bleeding events and transfusions was observed in placebo patients, but the differences between the ≤2 and ≥3 subgroups did not reach statistical significance.

**Table 4:** Summary of adverse events by sequence of enrollment (ADDRESS)

Variable	Drotrecogin alfa (activated)			Placebo		
	
	≤2nd patient (n = 459)	≥3rd patient (n = 858)	*P *value	≤2nd patient (n = 422)	≥3rd patient (n = 851)	*P *value
Patients with ≥1 SBE	16 (5.5%)	35 (4.1%)	0.60	11 (2.5%)	17 (2.0%)	0.57
Patients with ≥1 SBE during infusion	9 (2.0%)	22 (2.6%)	0.49	6 (1.4%)	9 (1.1%)	0.63
Patients with ≥1 BE	61 (13.3%)	82 (9.6%)	0.04	33 (7.5%)	50 (5.9%)	0.27
Patients with ≥1 of any BE during infusion	53 (11.5%)	69 (8.0%)	0.04	28 (6.3%)	41 (4.8%)	0.25
Patients requiring any blood transfusion	38 (8.3%)	52 (6.1%)	0.13	19 (4.3%)	25 (2.9%)	0.20
Patients with ≥1 BE and who did not survive	28 (6.1%)	29 (3.4%)	0.02	7 (1.7%)	16 (1.9%)	0.68

'≤2nd patient' refers to the first two patients enrolled. '≥3rd patient' refers to the third and subsequent patients enrolled. Frequencies were analyzed using a chi-square test. ADDRESS, ADministration of DRotrecogin alfa (activated) in Early Stage Severe Sepsis; BE, bleeding event reported as an adverse event; SBE, bleeding event reported as a serious adverse event.

Mortality for patients experiencing any bleeding event in ADDRESS was higher than for patients not experiencing a bleeding complication, irrespective of treatment group. For placebo patients, 28-day mortality rates were 27.7% (95% confidence interval [CI] 18.1% to 37.3%) and 16.3% (95% CI 14.2% to 18.4%) for patients who did and did not experience a bleeding complication, respectively. For DrotAA, 28-day mortality rates were 40.1% (95% CI 32.1% to 48.2%) and 15.8% (95% CI 13.8% to 17.9%) for patients who did and did not experience a bleeding complication, respectively. The percentage of patients who experienced a bleeding event and subsequently died was significantly less for DrotAA patients in the ≥3 subgroup compared with the 2 subgroup (3.4% versus 6.1%; *P *= 0.02), whereas there was no difference between subgroups in placebo patients (1.9% versus 1.7%; *P *= 0.68) (Table 4). An analysis of baseline characteristics for patients who experienced any bleeding event indicated that these patients were more severely ill compared with patients who did not have a bleeding event (data not shown). Consequently, the presence of a bleeding complication could have been a marker for higher disease severity; hence, these patients might have been expected to have higher mortality.

### Sequence effect in selected subgroups

**Figure 2:**
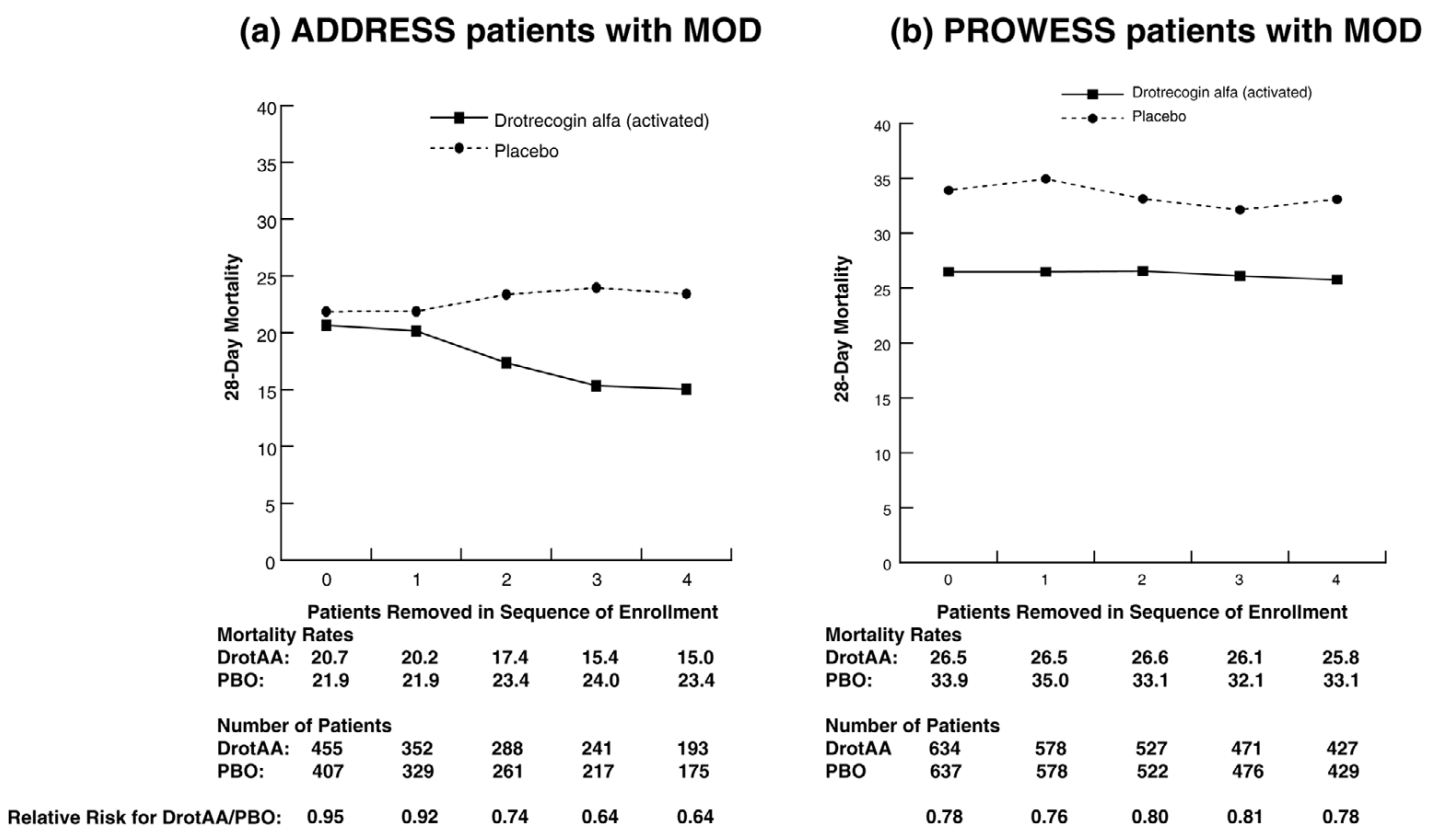
Twenty-eight-day mortality in all randomly assigned patients with multiple organ dysfunction in ADDRESS **(a) **and PROWESS **(b) **with no patients removed from the analysis and also with the first through fourth patients enrolled at each site removed. Note: 0 represents the results for the entire population, and 1 through 4 correspond to the analysis with the first through fourth patients from each site removed. ADDRESS, ADministration of DRotrecogin alfa (activated) in Early Stage Severe Sepsis; DrotAA, drotrecogin alfa (activated); MOD, multiple organ dysfunction; PBO, placebo; PROWESS, Protein C Worldwide Evaluation in Severe Sepsis.

**Figure 3:**
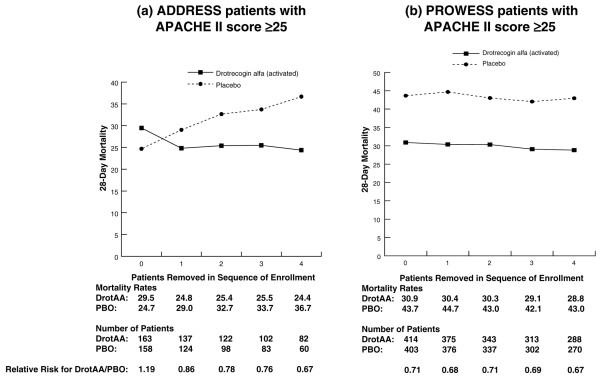
Twenty-eight-day mortality in all randomly assigned patients with an APACHE II score of greater than or equal to 25 in ADDRESS **(a) **and PROWESS **(b) **with no patients removed from the analysis and also with the first through fourth patients enrolled at each site removed. Note: 0 represents the results for the entire population, and 1 through 4 correspond to the analysis with the first through fourth patients from each site removed. ADDRESS, ADministration of DRotrecogin alfa (activated) in Early Stage Severe Sepsis; APACHE, Acute Physiology and Chronic Health Evaluation; DrotAA, drotrecogin alfa (activated); PBO, placebo; PROWESS, Protein C Worldwide Evaluation in Severe Sepsis.

As statistically significant treatment-by-sequence of enrollment interactions were present in both ADDRESS and PROWESS, selected subgroups from both studies were also examined. Figure 2 displays 28-day mortality for patients with MOD enrolled in ADDRESS or PROWESS for subgroups in which the first through fourth patients enrolled at each site were excluded from the analysis. A treatment-by-sequence of enrollment interaction was present in the ADDRESS (*P *= 0.006) but not in the PROWESS MOD subpopulations. A similar analysis was performed for the APACHE II score ≥25 subgroup (Figure 3). As with the MOD subgroup in PROWESS, no treatment-by-sequence of enrollment interaction was present in PROWESS patients with APACHE II scores ≥25. However, in the ADDRESS subgroup, we observed a significant interaction (*P *= 0.01) that appeared to result from an increase in the mortality rate for placebo patients, despite the absence of an increase in the APACHE II score as sites enrolled more patients. There was not a parallel increase in mortality for the DrotAA patients.

**Figure 4:**
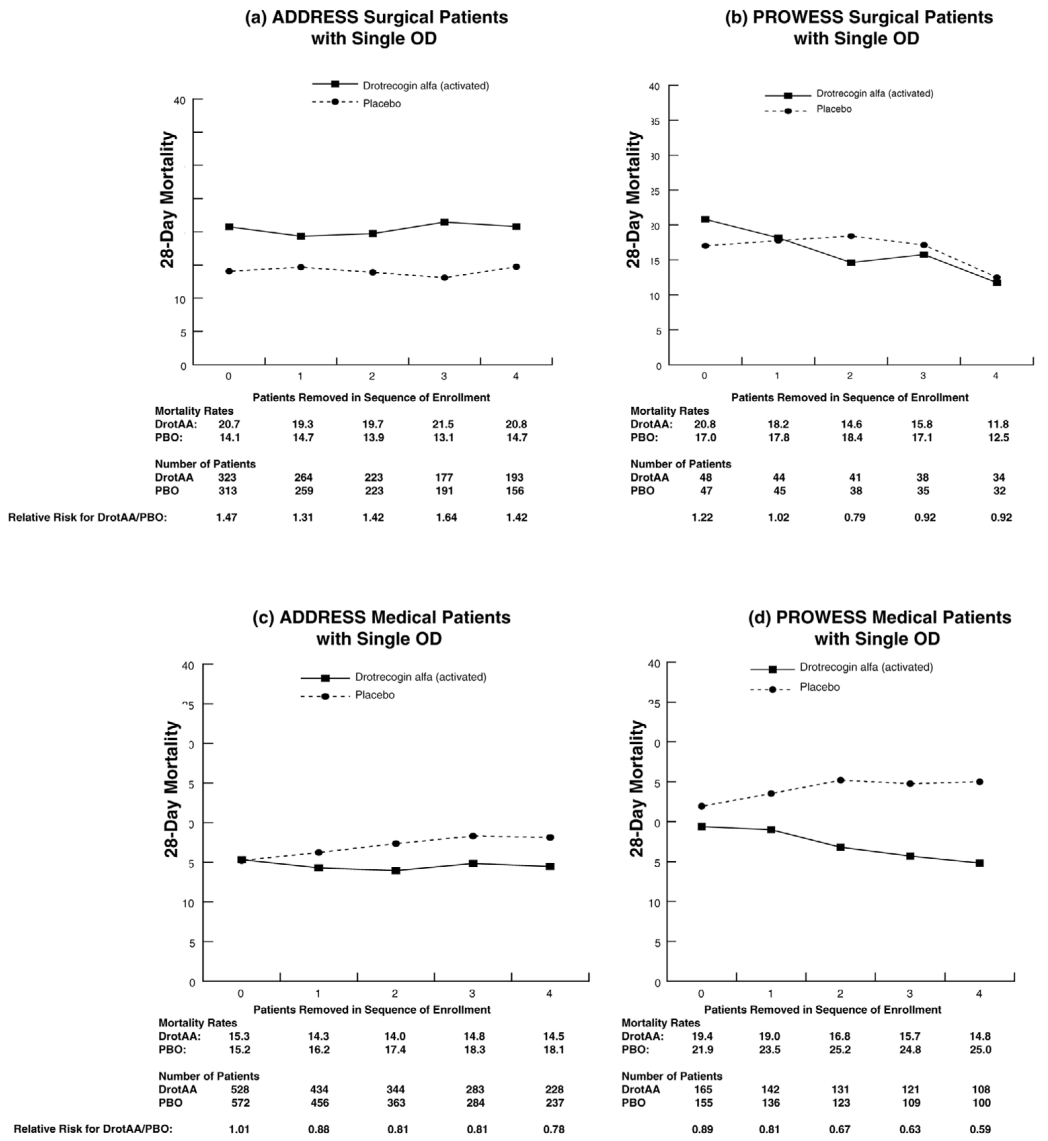
Twenty-eight-day mortality in all randomly assigned surgical patients with single organ dysfunction in ADDRESS **(a) **and PROWESS **(b) **with no patients removed from the analysis and also with the first through fourth patients enrolled at each site removed. Note: 0 represents the results for the entire population, and 1 through 4 correspond to the analysis with the first through fourth patients from each site removed. ADDRESS, ADministration of DRotrecogin alfa (activated) in Early Stage Severe Sepsis; DrotAA, drotrecogin alfa (activated); OD, organ dysfunction; PBO, placebo; PROWESS, Protein C Worldwide Evaluation in Severe Sepsis.

**Figure 5:**
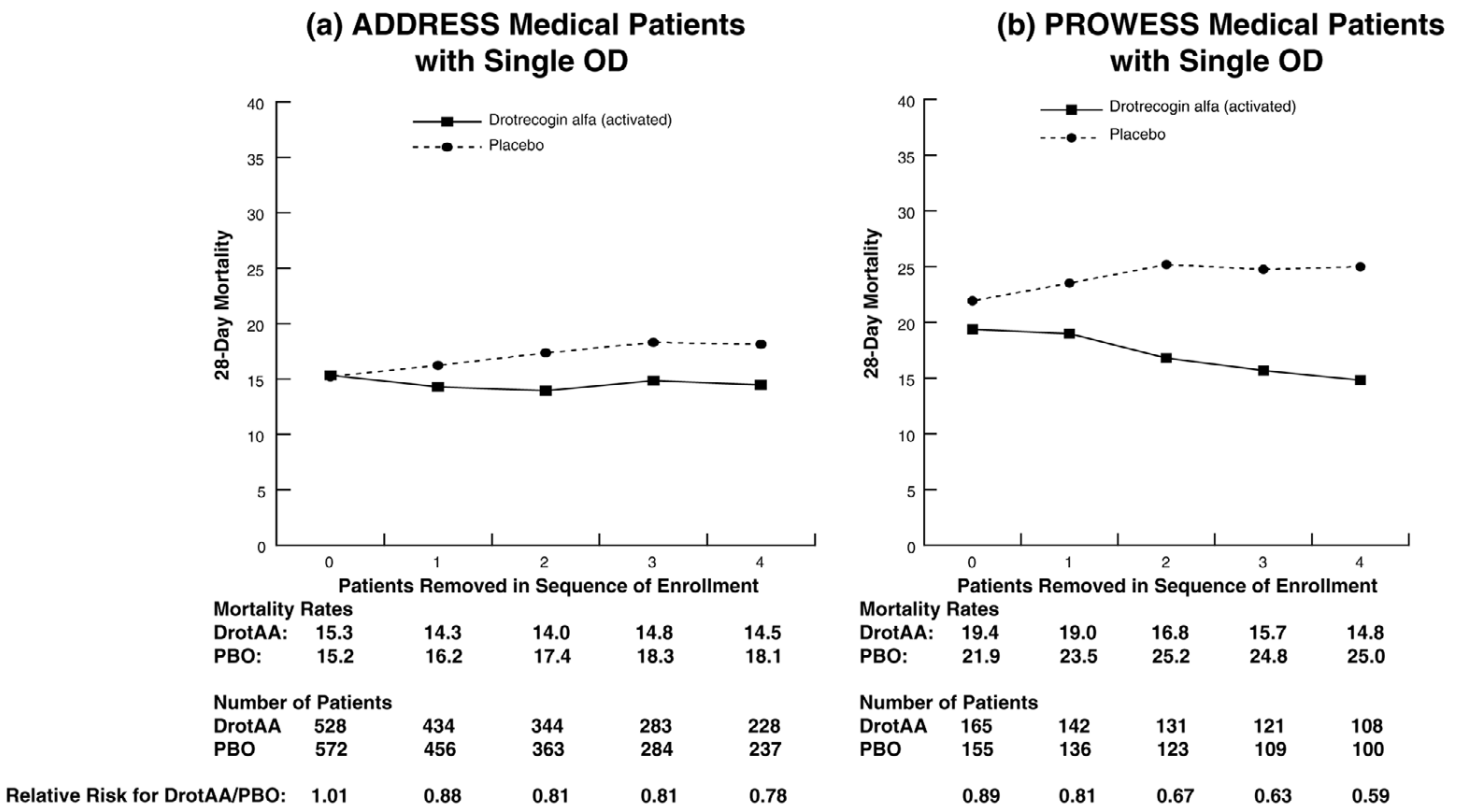
Twenty-eight-day mortality in all randomly assigned medical patients with single organ dysfunction in ADDRESS **(c) **and PROWESS **(d) **with no patients removed from the analysis and also with the first through fourth patients enrolled at each site removed. Note: 0 represents the results for the entire population, and 1 through 4 correspond to the analysis with the first through fourth patients from each site removed. ADDRESS, ADministration of DRotrecogin alfa (activated) in Early Stage Severe Sepsis; DrotAA, drotrecogin alfa (activated); OD, organ dysfunction; PBO, placebo; PROWESS, Protein C Worldwide Evaluation in Severe Sepsis.

**Figure 6:**
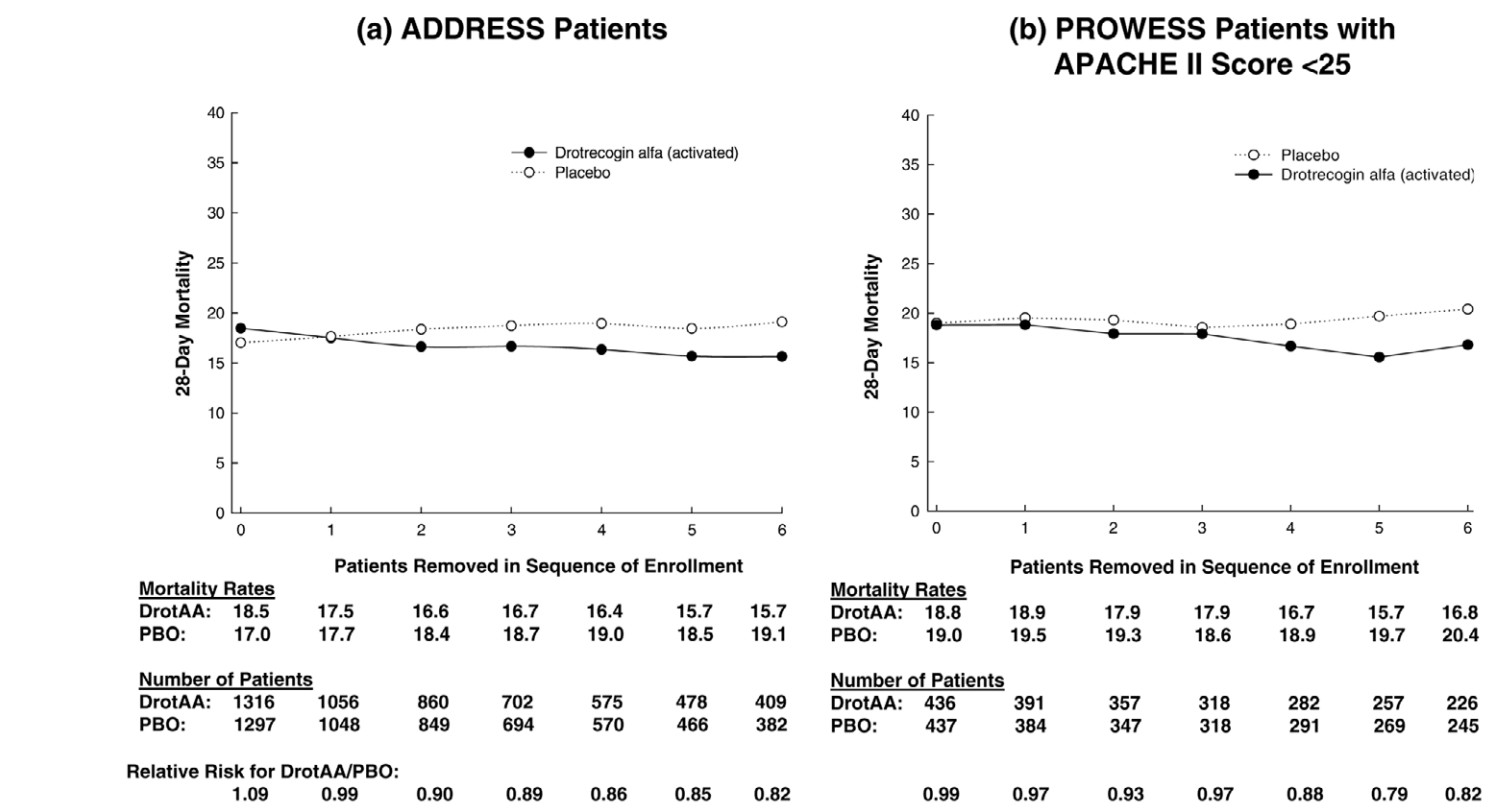
Twenty-eight-day mortality in all randomly assigned patients in ADDRESS **(a) **and in PROWESS **(b) **with an APACHE II score of less than 25, with no patients removed from the analysis, and also with the first through fourth patients enrolled at each site removed. Note: 0 represents the results for the entire population, and 1 through 4 correspond to the analysis with the first through fourth patients from each site removed. ADDRESS, ADministration of DRotrecogin alfa (activated) in Early Stage Severe Sepsis; APACHE, Acute Physiology and Chronic Health Evaluation; DrotAA, drotrecogin alfa (activated); PBO, placebo; PROWESS, Protein C Worldwide Evaluation in Severe Sepsis.

Similar analyses were conducted for subpopulations defined as single-organ dysfunction surgical patients (Figure 4) and single-organ dysfunction medical patients (Figure 5). For single-organ dysfunction surgical patients, no treatment-by-sequence of enrollment interaction was present in either the ADDRESS or the PROWESS study. However, mortality was higher for DrotAA patients compared with placebo patients in ADDRESS (20.7% versus 14.1%, n = 636) and was similar to placebo patients in PROWESS (Figure 4). In ADDRESS, an analysis by type of single-organ dysfunction in surgical patients suggested that the higher mortality observed in DrotAA compared with placebo patients was due to those patients with isolated respiratory failure (21.1% versus 10.4%; *P *< 0.05). Strong trends for treatment interactions were present in the single-organ dysfunction medical patients in both the ADDRESS (*P *= 0.051) and PROWESS (*P *= 0.058) trials (Figure 5). As the above results suggested that any treatment-by-sequence of enrollment interaction might be most apparent in lower-risk patients, the entire ADDRESS population was compared with the lower-risk population in PROWESS as defined by an APACHE II score of less than 25 (Figure 6). A treatment-by-sequence of enrollment interaction was present in ADDRESS (*P *= 0.04) and only a trend in PROWESS (*P *= 0.11).

## Discussion

In both PROWESS and ADDRESS, there appeared to be an influence of enrollment sequence within a site on the observed treatment effect with DrotAA. Analyses of the ADDRESS and PROWESS studies suggest that the enrollment sequence effect or 'learning curve' may be largely confined to the populations with a lower risk of death and may relate to difficulties in diagnosis, timely diagnosis, or a situation in which small absolute reductions in mortality may not overcome the complications of therapy.

In ADDRESS, as in the PROWESS study, there was a statistically significant interaction between the observed treatment effect associated with DrotAA and the sequence in which patients were enrolled at study sites. In both trials, greater mortality was found with DrotAA use within the subgroup that comprised the first patients enrolled at study sites. In PROWESS, mortality was lower with DrotAA treatment for the second and subsequent patients enrolled at a site. However, in ADDRESS, mortality remained higher with DrotAA compared with placebo for the second patients enrolled. Thereafter, mortality was lower for DrotAA-treated patients compared with placebo.

A comparison of baseline characteristics for patients in ADDRESS who comprised the first two patients enrolled at a site compared with the third and subsequent patients indicated that there were changes in the characteristics of patients enrolled in the study as the site gained experience with the protocol. Specifically, differences were observed between the subgroups in the racial origin of patients, acute physiology scores, percentage of patients with chronic health points, surgical status, and heparin use. The mean time from the onset of first organ failure to treatment with study drug decreased from 24.1 to 21.8 hours for the first two patients compared with subsequent patients (*P *< 0.001). The observed relative risk for DrotAA was similar between studies for patients enrolled in the third and subsequent patient blocks (0.82 versus 0.80 and 0.69 versus 0.69 for patients in the third block and subsequent blocks, respectively). An analysis of outcomes within clinically relevant subgroups suggested that any sequence effect is contained within the population of patients at lower risk of death. These data suggest that a potential sequence effect was present in the ADDRESS study as was observed in PROWESS.

An analysis of bleeding complications by sequence of enrollment in ADDRESS also indicated that sites enrolled patients at lower risk of bleeding in both the DrotAA and placebo groups. The percentage of patients experiencing any bleeding complications declined in both treatment groups as sites enrolled more patients into the study. Patients in the ≥3 subgroup who received DrotAA received fewer transfusions compared with those patients in the ≤2 subgroup. In both treatment groups, mortality was higher for patients experiencing any bleeding complications compared with those who did not have a bleeding event. As bleeding events were more common in the DrotAA groups, there were more patients in this group compared with placebo who experienced any bleeding event and did not survive. Patients experiencing a bleeding event were more severely ill than those who did not experience a bleeding event, so a direct contribution to the cause of death could not be determined but certainly cannot be excluded.

Determining the influence of sequential changes in patient characteristics on the outcome of ADDRESS is difficult. Previous data have suggested that the observed treatment effect associated with DrotAA is larger if treatment is initiated within 24 hours of first organ failure [8]. The presence of a sequence effect in low-risk patients only may suggest the difficulty of making the diagnosis of severe sepsis in this clinical setting. The influence of protocol violations on outcome could not be assessed because less than 50% of patients were monitored. However, protocol violations reduced the observed treatment effect in PROWESS [3]. Fewer bleeding complications and transfusions in DrotAA patients comprising the third and subsequently enrolled subgroup could have contributed to the lower observed mortality. However, an important aspect of these data is that the population of patients enrolled in a clinical study may change as sites gain experience with a protocol and patient identification, leading to change in the observed treatment effect associated with an intervention as sites enroll more patients in the study. These observations have important implications for the design of future trials. For example, the ongoing PROWESS shock study (a randomized placebo-controlled trial of DrotAA in adults with persistent shock) is using an academic coordinating center to approve every patient enrolled into the study. In addition, this trial has robust source data verification and documentation of protocol violations which will allow for re-education if the sites demonstrate inadequate understanding of the protocol.

Learning curves have been described in other clinical trials and in clinical practice. Halm and colleagues [9] performed a literature review of studies examining the independent relationship between hospital or physician volume and clinical outcomes. They found that, overall, 71% of all studies of hospital volume and 69% of studies of physician volume reported statistically significant associations between higher volume and better outcomes. The strongest associations were found for AIDS treatment and for surgery on pancreatic cancer, esophageal cancer, abdominal aortic aneurysms, and pediatric cardiac problems. However, it is not always clear what exactly is responsible for these different outcomes.

While it is perhaps easier to explain a sequence effect in relation to surgical procedures, as well as AIDS treatments noted earlier, there have been a number of studies investigating the relationship between outcome and clinical experience with the use of tissue-type plasminogen activator (tPA) in ischemic stroke. Heuschmann and colleagues [10] found that data from the German Stroke Registers Study Group indicated that in-hospital mortality of ischemic stroke patients after tPA use varied among hospitals with different experience in tPA treatment in routine clinical practice. There have been additional studies from different countries indicating that tPA should be administered by experienced physicians in hospitals with expertise in acute stroke care and that published treatment guidelines should be followed [11-15]. Similar to the findings associated with following tPA treatment guidelines, adherence to the American College of Critical Care Medicine-Pediatric Advanced Life Support guidelines for hemodynamic support for newborns and children in septic shock has been noted to be associated with improved survival [16].

Data analyses based on secondary objectives in a clinical study and *post hoc *exploratory analyses suffer from concerns over the play of chance in multiple comparisons as well as reporting bias. Primary and secondary analyses have been reported for both the PROWESS and ADDRESS studies. The initial report of a treatment-by-enrollment interaction in PROWESS was considered hypothesis-generating. However, this finding in a subsequent trial suggests that such a sequence effect may have important implications for study design and data interpretation. Nevertheless, such findings do not alter the overall conclusions of these studies.

The exact cause of the 'enrollment sequence' remains uncertain. We have demonstrated associated changes in patient characteristics, but it is not clear whether these or other 'unrecorded' differences may be responsible. Changes in patient management, particularly in relation to DrotAA administration, could also be important, but not all of the details of patient management were collected. Two factors that could be important are time to treatment, which has previously been linked to improved outcome with DrotAA treatment [17], and the frequency of bleeding events. We hypothesize that the 'sequence effect' may be more apparent in populations with lower mortality risk because the absolute mortality reduction is anticipated to be less in this population and thus it may be more difficult to demonstrate efficacy in this situation unless treatment conditions are optimized. The 'enrollment sequence' may thus have been more apparent in ADDRESS because much of this population was at low risk.

## Conclusion

Analysis of the ADDRESS and PROWESS studies suggests that an enrollment sequence effect was present in both studies. In PROWESS, this enrollment sequence effect reduced the observed treatment effect associated with DrotAA. In ADDRESS, this enrollment sequence effect may have contributed to the decision to recommend early termination of the study for futility. The finding of such an enrollment sequence effect in two separate clinical trials in severe sepsis suggests that trial designs, site training, data collection and monitoring, and statistical analysis plans need to be adjusted for these potential confounders. A list of potential recommendations to assist in the design of future clinical trials is shown in Table 5.

**Table 5:** Potential recommendations to assist in the design of future clinical trials

Recommendation	Potential benefits	Potential issues
More extensive inclusion and exclusion criteria that are more descriptive of the population to be enrolled	Less opportunity for patient variability and sites having to 'learn as they go'	Lower likelihood of extrapolating efficacy observed in the clinical trial to effectiveness in clinical practice

Standardize the major facets of severe sepsis concomitant care	Reduced variability as caring for patients with severe sepsis may be a more complex 'procedure' than many commonly performed surgical procedures	May be questions related to the applicability of the study results to a more general severe sepsis population, in which concomitant care has not been standardized

Given the heterogeneity of severe sepsis patients, different populations of patients may require unique sets of inclusion and exclusion criteria (for example, medical patients and surgical patients).	Optimizes inclusion and exclusion criteria without the extra time and resources that would be needed to run two separate studies	May be issues with interpretation of data and powering if the treatment effect differs significantly between the two populations, in which two separate studies may be preferable

Use a clinical coordinating center to assist study sites in enrollment of eligible patients.	Helps to optimize protocol compliance	May be questions related to the applicability of the study results to a more general severe sepsis population

Site selection should be based on having good clinical trial and critical care experience.	Helps to minimize variability and (potentially) protocol violations	May be questions related to the applicability of the study results to a more general severe sepsis population

The use of severity scoring systems in clinical trials may require training and validation of the training to ensure proper collection of severity of illness information.	Helps to ensure the collection of accurate data	Additional time and resources required

Given the potential influence of site experience on outcomes, randomization stratified by site should be considered a requirement for studies in complex disease states.	Helps to minimize effect of differences between sites and enrollment sequence effect	May limit ability to stratify by additional parameters

Planned enrollment per site should be based on the block size used for randomization. Expected enrollment per site should be at least two full blocks of patients.	Helps to minimize enrollment sequence effect	May only be able to have larger sites in the study, raising questions related to generalizability of the results

Futility stopping rules should incorporate the potential for learning curves to obscure a beneficial treatment effect in the early stages of a clinical trial.	Helps to avoid type II error	May be a delay in identifying futility signals if no enrollment sequence effect is present

Statistical analysis plans should explore the potential for learning curves within the clinical trial dataset.	Prospectively defined analyses have greater weight and may help to explain study findings	Additional workload

Clinical studies should have a prospectively defined monitoring plan. Source data verification and documentation of protocol violations should be performed on the first few patients enrolled at a site until the site demonstrates adequate understanding of the protocol.	Helps to minimize protocol violations	Additional time and resources required

## Key messages

• In the ADDRESS and PROWESS studies, a significant interaction between drotrecogin alfa (activated) treatment effect and the sequence in which patients were enrolled was observed.

• Characteristics of patients enrolled first changed compared with subsequent patients, including earlier treatment and fewer protocol violations in the latter group.

• The enrollment sequence effect observed in two separate trials suggests the need for analyses adjusted for confounding events.

• Future study designs in severe sepsis should include site selection and training, close monitoring of strict protocol adhesion, sufficient recruitment per site, and a clinical coordinating center to ensure adequate patient selection.

## Abbreviations

ADDRESS: Administration of Drotrecogin Alfa (Activated) in Early Stage Severe Sepsis; APACHE: Acute Physiology and Chronic Health Evaluation; CI: confidence interval; DrotAA: drotrecogin alfa (activated); MOD: multiple-organ dysfunction; PROWESS: Protein C Worldwide Evaluation in Severe Sepsis; tPA: tissue-type plasminogen activator.

## Competing interests

Eli Lilly and Company provided financial support for this study. P-FL, ARJG, J-FD, and EA have participated in trials sponsored by Eli Lilly and Company and/or have served as paid consultants for Eli Lilly and Company. WLM, JJ, MDW, and DRN are employees and stockholders of Eli Lilly and Company.

## Authors' contributions

P-FL and EA participated in the conception and design of the study, in the development and conduct of analyses, and in the clinical trials and data collection. WLM, JJ, and DRN participated in the conception and design of the study, in the development and conduct of analyses, and in drafting the manuscript. MDW participated in drafting the manuscript. J-FD participated in the clinical trials and data collection. All authors contributed to the analysis and interpretation of the data and to critical review, revisions, and final approval of the final manuscript.
